# Differential induction of inflammatory cytokines by dendritic cells treated with novel TLR-agonist and cytokine based cocktails: targeting dendritic cells in autoimmunity

**DOI:** 10.1186/1476-9255-7-37

**Published:** 2010-07-27

**Authors:** Simon S Jensen, Monika Gad

**Affiliations:** 1 Department of immune targeting, Bioneer A/S, Kogle Allé 2, Hørsholm, DK-2970, Denmark; 2 Bioneer A/S, Kogle Allé 2, Hørsholm, DK-2970, Denmark

## Abstract

**Background:**

Dendritic cells (DC) are main gate-keepers of the immune system, bridging the innate and adaptive immune system. DCs are able to mature into inflammatory DCs at sites of inflammation in both autoimmune and allergic disease, thereby sustaining a continuous activation of the adaptive immune system at sites of inflammation. This function of DCs makes them attractive target cells for therapeutic intervention in inflammatory diseases. We have designed a DC-based screening model by which drug candidates can be evaluated for their ability to suppress DC maturation into an inflammatory and disease promoting phenotype.

**Methods:**

Human monocyte derived DCs were differentiated using IL-4 and GM-CSF to immature DCs (imDCs). The imDCs were treated with various combinations of TLR-agonists and pro-inflammatory cytokines to identify cocktails with ability to mature imDCs into inflammatory DCs. The effect of the cocktails on DC maturation was evaluated using ELISA and cytokine arrays to measure secreted cytokines and chemokines. FACS analysis was used to assess expression of maturation markers, and functional studies were carried out using naïve allogeneic T-cells to assay for a Th1-promoting DC phenotype.

**Results:**

Nine cocktails were designed with potent ability to induce secretion of the Th1-promoting cytokines IL-12p70 and TNFα from imDCs, and three were able to induce the Th17-promoting cytokine IL-23. The cocktails were further characterized using cytokine arrays, showing induction of inflammation related cytokines and chemokines like CXCL10, CCL2, CCL4, CCL8, CCL15, CCL20 and IL-8, of which some are present in a range of autoimmune pathologies. Prostaglandin E2 secretion was identified from DCs treated with TLR agonists poly I:C and peptidoglycan, but not LPS. The cocktails were able to induce DC maturation markers like HLA-DR, CD40, CD80, CD83 and CD86, except the TLR7/8 agonist R848. Functional end-points made by co-culture of allogeneic CD4^+ ^T cells with the cocktail treated DCs, showed that five cocktails in particular could induce a classical Th1-phenotype with ability to secrete high amounts of the hall-mark cytokine IFNγ. The model was validated using dexamethasone and two COX-inhibitors, which were able to suppress the cocktail driven pro-inflammatory DC maturation.

**Conclusions:**

The identification of novel Th1-promoting cocktails allows screening of anti-inflammatory drug candidates by assessing the ability to suppress the activation and differentiation of imDCs into inflammatory DCs with a specific Th1-promoting phenotype. The model thus provides a screening tool, which can identify potential anti-inflammatory effects on the natural regulator of the immune response, the dendritic cell.

## Background

Dendritic cells (DCs) are central in the pathogenesis of immune disorders, where they respond towards environmental factors by regulating the adaptive immune system through activation and expansion of T cells. In autoimmunity the immature DCs develop into inflammatory DCs, which present self-antigens to T cells, which are activated towards the self-antigen, causing an autoimmune response. The inflammatory DCs are responsible for secretion of cytokines with a pro-inflammatory function like TNFα, IL-12p70, IL-23, and several other inflammatory mediators like nitric oxide, prostaglandins and chemokines [1,2]. During inflammation, immature DCs are attracted to the site of inflammation by chemokines in the microenvironment, and a large number of DCs are often present at sites of inflammation. After antigen uptake at the inflammatory site and maturation by inflammatory cytokines and chemokines, the DCs differentiate into inflammatory DCs which migrate to the lymph nodes and stimulate T cell function and proliferation. Since DCs have a short half-life under inflammatory conditions and are upstream in the inflammatory process, they are attractive target cells for therapeutic intervention of inflammatory diseases [1]. DCs express a unique repertoire of receptors essential for the innate immune response, termed pattern recognition receptors (PRRs), including the Toll Like Receptors (TLRs), nucleotide binding and oligomerization domain-like receptors (NLRs) and C-type lectin-like receptors (CLRs) [3]. These receptors and their corresponding signalling pathways are involved in the pathology of autoimmune diseases like e.g. psoriasis and rheumatoid arthritis (RA), and in particular inflammatory bowel disease (IBD) defining Crohns disease and ulcerative colitis [4]. DCs also express receptors involved in cytokine responses as well as chemokine receptors involved in cell migration, and are thus responsive to various types of environmental factors [5]. Pro-inflammatory activation of DCs can be naturally counterbalanced by inhibitory molecules believed to regulate and fine-tune T-cell responses, and particular the B7-H1 and H4 receptors provide negative signals that suppress effector T-cell responses [6]. Finally, DCs are the source of secretion of very potent pro-inflammatory cytokines and chemokines. The hall-mark cytokines involved in initiation of the adaptive Th1 immune responses are IL-12p70 whereas IL-6, TGFβ and IL-23 are involved in initiation and sustainment of Th17 differentiation. Both types of immune responses are known to be involved in chronic autoimmune disorders like e.g. Crohns disease [7,8]. Essential chemokines secreted from DCs are IL-8, CCL17, CCL18, CCL22 and APRIL, involved in both Th1 and Th2 type responses [8,9]. The unique and very complex signalling network in DCs involving PRRs and secretion of early triggers of inflammation, which are mainly associated with or restricted to DC function, opens a window of opportunities for DC-specific therapeutics in treatment of inflammatory disease [1]. The aim of this work was to mimic the development *in vitro*, of immature DCs into inflammatory DCs as seen in autoimmune conditions like e.g. Crohns disease, arthritis and psoriasis. We used human monocyte derived imDCs for evaluation of various combinations of TLR agonists and pro-inflammatory cytokines, in the attempt to design cocktails able to stimulate an inflammatory DC phenotype, mimicking the situation *in vivo*, where immature DCs migrate to sites of inflammation, after subsequent exposure to TLR agonists and inflammatory cytokines as seen in the inflammatory tissue. To mimic the potential therapeutic situation *in vivo*, where monocytes and imDCs are exposed to a drug prior to migration into the tissue and towards the inflammatory site, imDCs are in our model exposed to the drug candidate prior to exposure to the cocktail which mimics the microenvironment present at the inflammatory site. The ability of the drug and cocktail treated DCs to stimulate an immune response are then determined after 18-24 h, at a time point where inflammatory DCs *in vivo *are in the process of migration to and activation of the adaptive immune response in the lymph nodes.

We suggest that DC-based *in vitro *models of inflammatory conditions as described here are suitable models for screening of compounds or immune modulating agents like microorganisms specifically targeting immune disorders.

## Methods

### DC development from monocytes

PBMC were purified from buffy coats from healthy donors above the age of 18, which did not suffer from immune disorders or had been on recent medication. PBMCs were purified by centrifugation over a Ficoll-pague (GE Healthcare limited, Buckinghamshire, UK) gradient, and monocytes were isolated from PBMCs by positive selection of CD14^+ ^cells (specific for monocytes) by magnetic beads (Dynal, Invitrogen, Carlsbad, CA, USA) according to the manufacturer's instructions. The CD14^+ ^monocytes were cultured in 6-well plates in a conc. at 2 × 10^6 ^cells/ml (3 mL/well) in RPMI/5% FCS supplemented with recombinant human GM-CSF (20 ng/mL) and IL-4 (20 ng/mL), (PeproTech, London, UK). The medium was changed after 2 and 3 days. After 6 days of culture the immature DCs were re-cultured into 96-well plates at 10^6 ^cells/well.

### ELISA and PGE2 measurements

Human ELISA kits were used from the following manufacturers: IL-12p70, TNFα, (R&D Systems, Minneapolis, MN, USA), IFNγ, IL-23 (eBioscience, San Diego, CA, USA). Prostaglandin E2 was measured using the Prostaglandin E2 EIA Kit - Monoclonal (Cayman Chemical, Ann Arbor. MI, USA). DCs were setup in 96 well plates with 100.000 cells/well and cocktails tested in triplicates. After 24 h incubation with cocktails, the conditioned media was removed from the wells and stored at -80°C until analyses. The media was diluted to reach the linear range for each ELISA assay, and the amount of cytokine for each sample was determined in duplicate. Using a standard curve provided in the kit, the concentration of cytokines was determined for each sample.

### Cytokine array

Human inflammation antibody based cytokine arrays were used from RayBiotech, (Norcross GA, USA) according to manufacturers instructions, using conditioned media from DCs from four different donors treated with the individual cocktails and mixed in equal amounts. The membranes were developed using west pico luminescence reagent from Pierce (Rockford, IL, USA) and exposed on Amersham hyperfilm ECL, (GE Healthcare limited, Buckinghamshire, UK). Exposures which allowed identification of most abundantly secreted cytokines and chemokines were scanned and quantitated using the ImageJ-software from NIH, and induced cytokines and chemokines for each cocktail compared to the level seen for non-treated DCs. Induction up to 10 fold of the level seen for non-treated DCs was termed weakly induced, between 10-100 fold was termed modest induction, and above 100 fold induction termed strong induction. The induced cytokines and chemokines are shown in table 1. Constitutively secreted proteins as seen for non-treated DCs included IL-8, CCl2, CCL5, CCL13, TGFβ, TIMP1 and 2.

**Table 1:** Cocktail compositions and the cocktail stimulated DC expression pattern for selected cytokines and chemokines on cytokine arrays

Cocktail	Composition	Secreted cytokines and chemokines		
		
		Weak induction (1-10 fold)	Modest induction (10-100 fold)	Strong induction (> 100 fold)
LPS	LPS (0,1ug/mL)	IL-8, CCL2, CCL4, CXCL5, CXCL10, GM-CSF	IL-10, CCL20, CXCL1-3	IL-6, IL-12, TNFα, VEGF, CCL8, CCL15, CXCL1

Cocktail 1	IFNγ (20ng/mL)+ TNFα (50ng/mL)+ Poly I:C 12,5 ug/mL + IL-1β (10ng/mL) + IFNα (6ng/mL)	IL-8, CCL2, CCL4, CCL5, CXCL5, CXCL10	IL-10, CCL20, CXCL1-3, GM-CSF	IL-6, IL-12, TNFα, VEGF, CCL8, CCL15, CXCL1

Cocktail 2	IFNγ (20ng/mL)+ Poly I:C 12,5 ug/mL + IL-1β (10 ng/mL) + IFNα (6ng/mL)	IL-8, CCL2, CCL4, CCL5, CXCL5, CXCL10	IL-10, CCL20, CXCL1-3, GM-CSF	IL-6, IL-12, TNFα, VEGF, CCL8, CCL15, CXCL1

Cocktail 3	IFNγ (20ng/mL)+TNFα (50ng/mL)+ peptidoglycan (10ug/mL)	IL-8, CCL2, CCL4, CCL5, CXCL5	IL-10, CCL20, CXCL1-3, CXCL10, GM-CSF	IL-6, IL-12, TNFα, VEGF, CCL8, CCL15, CXCL1

Cocktail 4	LPS (0,1ug/mL)+ IFNγ (20ng/mL)	IL-8, CCL2, CCL4, CCL5, CXCL5, GM-CSF	IL-10, CCL20, CXCL1-3, CXCL10	IL-6, IL-12, TNFα, VEGF, CCL8, CCL15, CXCL1

Cocktail 5	LPS (0,1ug/mL)+ IFNγ (20ng/mL)+ TNFα (50ng/mL)	IL-8, CCL2, CCL4, CCL5, CXCL5, GM-CSF	IL-10, CCL20, CXCL1-3, CXCL10	IL-6, IL-12, TNFα, VEGF, CCL8, CCL15, CXCL1

Cocktail 6	R848 (1,0ug/mL)	IL-8, CCL2, CCL4, CCL5, CXCL10	IL-10, CCL15, CCL20, CXCL1-3, CXCL5, GM-CSF	IL-6, IL-12, TNFα, VEGF, CCL8, CXCL1

Cocktail 7	R848 (1,0ug/mL) + IFNγ (20ng/mL)	IL-8, CCL2, CCL4, CCL5, CCL20, CXCL5, GM-CSF	IL-10, CCL15, CCL20, CXCL1-3, CXCL10	IL-6, IL-12, TNFα, VEGF, CCL8, CXCL1

Cocktail 8	R848 (1,0ug/mL) + poly I:C (10ug/mL)	IL-8, CCL2, CCL4, CCL5, CXCL5, CXCL10	IL-10, CCL20, CXCL1-3, GM-CSF	IL-6, IL-12, TNFα, VEGF, CCL8, CCL15, CXCL1

Cocktail 9	IFNγ (20ng/mL) + IL-1β (10ng/mL)	IL-8, CCL2, CCL4, CCL5, CXCL5, GM-CSF	IL-10, CCL15, CCL20, CXCL1-3, CXCL10	IL-6, IL-12, TNFα, VEGF, CCL8, CXCL1

Table 1 shows the composition of cocktails combined by TLR-agonists and cytokines. TLR agonists were peptidoglycan (TLR2), Poly I:C (TLR3), LPS (TLR4/CD14/MD2), R848 (TLR7/8) and cytokines IFNγ, TNFα, IL-1β, IFNα. Secreted cytokines and chemokines were determined by combination of conditioned media for treatment of immature DCs from four different donors in order to obtain information for a general response and reduce single donor-variations. Signal intensities were semi-quantitatively calculated using the ImageJ software, and the fold of induction compared to untreated cells. Induced cytokines and chemokines were classified into weakly induced proteins (1-10 fold induced vs untreated), modestly induced proteins (10-100 fold induced vs untreated controls), and strongly induced proteins (more than 100 fold vs untreated controls).

### DC cocktail screening and validation

Potent IL-12p70 and TNFα stimulating cocktails were designed by series of combinations of pro-inflammatory cytokines and TLR agonists. The cytokines TNFα, IFNγ, IFNα IL-6 and IL-1β were from Peprotech (London, UK) and diluted in cell media to reach the indicated concentrations in table 1. TLR agonists were of TLR grade and from the following suppliers: lipopolysaccharide (LPS), polyinosinic polycytidylic acid (poly I:C), (Sigma, St. Louis, MI, USA). Peptidoglycan and R848 (Resiquimod) were from Alexis biochemicals (Axxora, San Diego, CA, USA). Cocktails were combined in a 5× working stock and added to immature DCs 30-60 min after counting and plating in 96 well plates. Cell density were 100.000 cells/96 well. For inhibition experiments, dexamethasone was added (0.01-0.1-1 uM) either 6 or 24 h prior to addition of cocktail to allow equilibrium and inhibition of inflammatory targets within the cells prior to addition of cocktails. The unspecific Cox inhibitor indomethacin (Sigma) was solubilized in ETOH, the specific Cox2 inhibitor NS398 (Cayman Chemical) was solubilized in DMSO and both added two h prior to cocktails. The DCs were routinely tested for cell viability, and no significant differences were seen for dexamethasone and/or cocktail treated cells (data not shown).

### Flow cytometric analysis

Harvested DCs were washed twice with PBS supplemented with 1% FBS. Fc receptors were blocked with excess human IgG (Sigma) on ice for 10 min. Immunofluorescence staining was performed by incubation of DCs for 30 min at 4°C with each mAb diluted to the optimal concentration according to the manufacturer's instructions. The following mAbs were used: anti-CD1a-APC, anti-CD14-Pe, anti-HLA-DR-Pe, anti- CD80-Pe, anti-CD83-Pe, anti-CD86-Pe, anti-CD40-Pe (all antibodies were from Becton Dickinson Pharmingen, NJ, USA). Relevant isotype controls were always used. Samples were acquired on a FACSArray (Becton Dickinson, NJ). At least 5000 mononuclear cells were gated using a combination of forward-angle and side scatter to exclude dead cells and debris. Data were analysed with FACSDiva software.

### Western blot

Cox2, and actin expression was analysed by Western blot as described by the supplier (Invitrogen). Briefly, DCs were seeded in 6 well plates at a density of 3-4 × 10^6 ^cells/well. 24 h after addition of cocktail, adherent and non-adherent cells were washed in ice cold PBS. Adherent cells were scraped off using a cell scraper and spun down. Loading buffer was added to the cell pellet and lysates processed as described by the manufacturer using Tris-Glycine based SDS-PAGE gels (Invitrogen). Exposure was made on ECL films as for cytokine arrays. Antibodies against Cox2 and actin were from Santa Cruz (Santa Cruz, CA, USA).

### MLR

In a MLR, DCs and peripheral blood lymphocytes from two different (allogeneic) individuals are mixed together in tissue culture round bottom wells for several days. DCs will stimulate lymphocytes from an incompatible individual to proliferate significantly whereas those from compatible individuals will not. CD4^+ ^T cells were isolated directly from PBMCs with anti-CD4 Dynabeads and Detach-aBead (Dynal, Invitrogen,) according to the manufacturer's instructions. Mixed lymphocyte cultures were performed in quadruplicates in 96-well round-bottom microtiter plates. A fixed number of 10^5 ^responding CD4^+ ^T cells were added to a 2 fold titrated number of allogeneic mitomycin-C treated (25 μg/ml for 30 min by 37°C) DCs starting from 10^4 ^(DC:T cell ratio: 1:10-1:640). The cells were cultured for 5 days and proliferation was measured after addition of 0.5 μCi/well of [^3^H]thymidine (Amersham, Little Chalfont, UK) for the last 18-24 h. The cells were harvested on a Filtermate 196 (Packard instruments, CT, USA) and incorporation was determined by liquid scintillation counting (Topcount, Packard Instruments, CT, USA).

### Analyses of cytokines secreted from T-cells in MLR

Supernatants from the mixed lymphocyte cultures were selected on day 5 and measured for levels of TNFα, IL-13 (R&D Systems) and IFNγ by ELISA (e-Bioscience). To further boost the secretion of cytokines, primed CD4^+ ^T cells from a 7 day MLR culture were collected and washed. For detection of cytokine production in the culture supernatants, the T cells were restimulated with plate-bound anti-CD3 (OKT3, 5 ug/mL) and soluble anti-CD28 (1 ug/mL) (both from BD Biosciences, NJ) at a concentration of 10^6 ^cells/mL for 24 h. The ELISA was performed in triplicates.

### Statistical analyses

Data were analyzed using unpaired, two sided t-test, (***P < 0.005, **P < 0.01, *P < 0.05).

## Results

### Identification of cocktails with the capability to induce secretion of the Th-1 promoting cytokines IL-12p70 and TNFα from human dendritic cells

**Figure 1:**
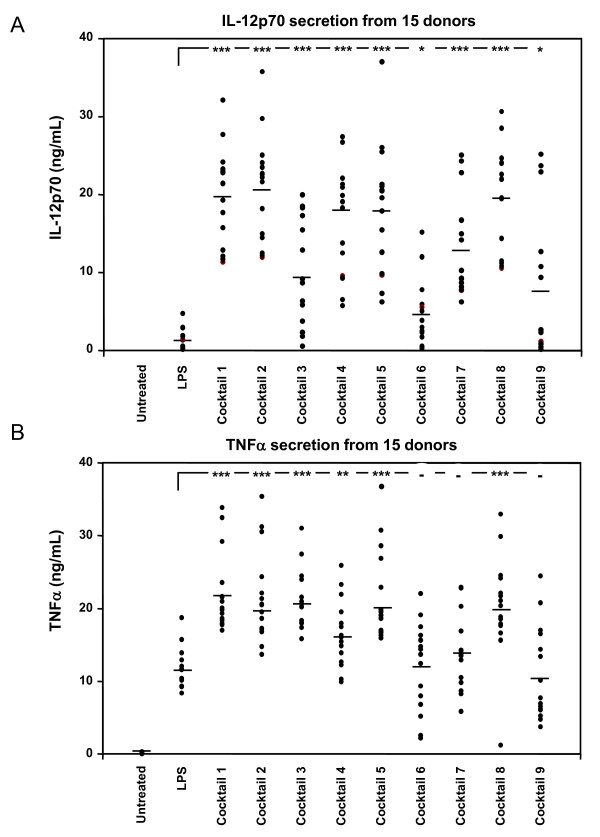
**Cocktail screening and donor variation for IL-12p70 and TNFα secretion**. A total of 15 different donor-derived imDCs were treated with LPS (0.1 μg/mL) or the 9 cocktails as indicated in table 1. After 24 h of incubation, IL-12p70 and TNFα levels in the conditioned media was determined by ELISA. A) The amount of IL-12p70 protein in each donor is indicated by a dot, and the average of all 15 donors indicated by a horizontal bar. B) Amount of TNFα protein was determined similarly in the 15 donors. Data were analyzed using unpaired, two sided t-test, (***P < 0.005, **P < 0.01, *P < 0.05).

The two pro-inflammatory cytokines IL-12p70 and TNFα were chosen as end-points for our present DC based screening model. After several rounds of optimization in order to select for potent combinations of TLR-agonists and cytokines, a range of cocktails were designed, which were able to induce IL-12p70 and TNFα secretion when added to immature DCs. The 9 most potent cocktails are seen in table 1, listed 1-9. In order to determine the variation in donor response for each cocktail, a series of 15 different donor-derived batches of DCs were tested for their individual response to the range of optimized cocktails from table 1. The secretion of IL-12p70 was determined for all DC-batches and the exact protein levels shown for each cocktail in figure 1A. LPS was included as a control in these experiments, but induced only slightly IL-12p70 compared to the optimized cocktails. The addition of peptidoglycan and poly I:C alone showed similar effects, with very weak induction of IL12-p70 (data not shown). The average IL-12p70 secretion ranged from approximately 5 ng/mL for cocktail 6 (R848), to above 20 ng/mL for cocktail 2 (IFNγ, Poly I:C, IL-1β, IFNα). The most potent IL-12p70 inducing cocktails are cocktail 1, 2, 4, 5 and 8. A similar analysis was made for secretion of TNFα, where the total amount of TNFα secreted for each cocktail treated DC batch is shown in figure 1B. The variation in total amounts secreted from each DC-batch was lower than for IL-12p70 secretion, indicating that the donor derived DCs are more responsive for induction of TNFα secretion than for IL-12p70 using these cocktails. LPS was able to induce TNFα secretion to significant levels in all DC-batches compared to untreated cells, in contrast to LPS induced IL-12p70 secretion. Cocktail 1 to 5 and 8 stimulated TNFα secretion significantly better than LPS, where cocktail 6, 7 and 9 did not.

### Cytokine array

**Figure 2:**
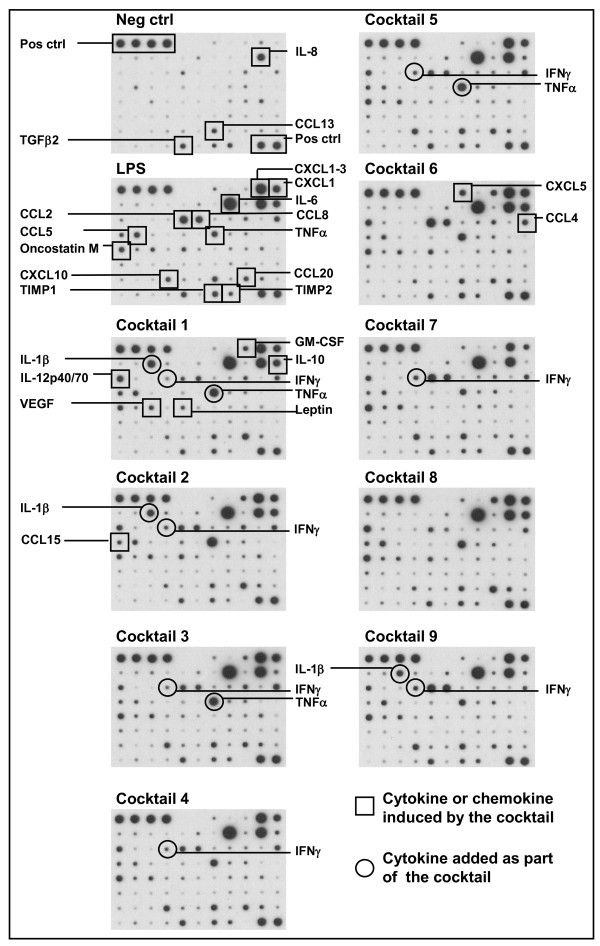
**Cytokine array using conditioned media from cocktail treated DCs**. Cytokine array membranes were incubated with conditioned media from DCs treated with cocktails for 24 h. To compensate for donor variations the conditioned media from four donors was mixed. Upper left four spots and lower right two spots serves as positive controls on each membrane. On the figure constitutively secreted cytokines can be seen on the picture indicated Neg. ctrl, which were untreated cells. Each induced protein is marked only once, squares mark DC-produced cytokines and chemokines, and a circle marks a cytokine which is added as part of the cocktail (TNFα, IL-1β and IFNγ).

In order to determine if the cocktails induce a cytokine and chemokine profile which corresponds to the pattern seen in tissue from patients suffering from Th1-directed immune disorders we performed a cytokine array on conditioned media from cocktail treated DCs mixed from four different donors (figure 2). LPS induced a range of pro-inflammatory proteins like IL-6, IL-8, TNFα, CCL2, 5, 8, 15, 20, CXCL1-3 and 10 (figure 2 and table 1). The cocktails all induced inflammation associated cytokines like IL-6 (> 100 fold), IL-12p70 (> 100 fold) and TNFα (> 100 fold). The cocktails also stimulated secretion of chemokines like IL-8, CXCL1-3, 5 and 10, CCL2, 4, 5, 8, 15, 20, which mainly are involved in recruitment of leukocytes like neutrophils, basophils, monocytes, DCs, Th1 and NK cells to sites of inflammation [10], as well as the angiogenic stimulator VEGF. Also the tolerance inducing cytokine IL-10 was induced by the cocktails, although not as strongly as IL-6, IL-12p70 and TNFα.

### Model validation using the anti-inflammatory drug dexamethasone

**Figure 3:**
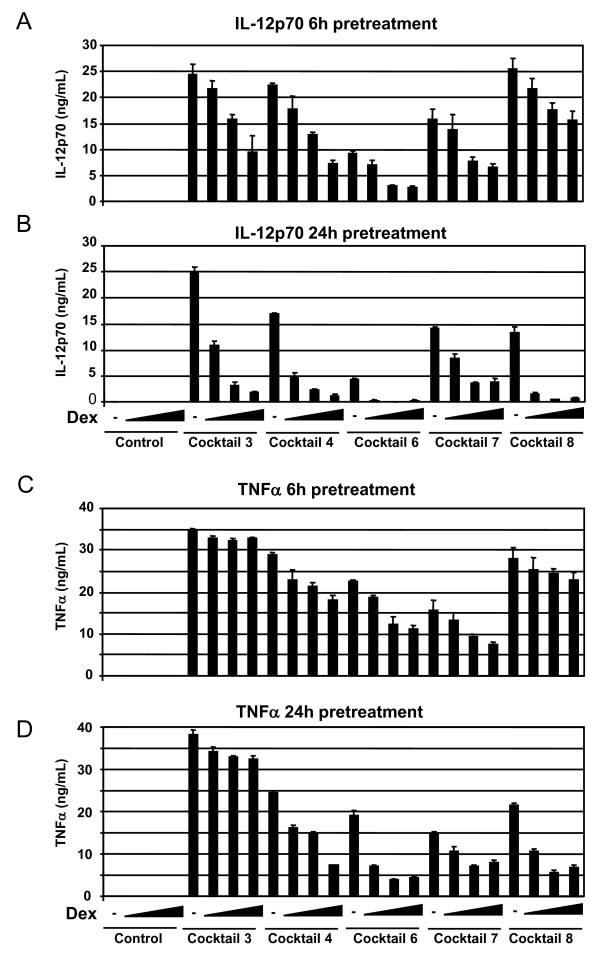
**Dexamethasone prevents cocktail induction of IL-12p70 and TNFα**. Immature DCs from a single donor shows a dexamethasone-mediated dose dependent suppression of IL-12p70 and TNFα secretion. Dexamethasone was pre-incubated with imDCs for 6 hours (A and C) or 24 hours (B and D) with increasing concentration of dexamethasone at 0-0.01-0.1 and 1.0 μM. Dexamethasone treatment without cocktail did not induce IL-12p70 or TNFα (first 4 bars). The cocktails used are indicated below each set of data, and their exact composition is seen in table 1. This shows one representative example out of three. Cell viability was not significantly affected by treatment with cocktail and/or dexamethasone (not shown).

The screening model was validated using dexamethasone (dex) to suppress the maturation of imDCs into inflammatory DCs. Dexamethasone was added to the imDCs for 6 or 24 h prior to addition of selected cocktails for another 24 h, with subsequent measurements of IL-12p70 and TNFα in the conditioned media (figure 3). Dexamethasone was able to prevent IL-12p70 secretion stimulated by cocktails 3, 4, 6, 7 and 8 in a dose-dependent manner. The suppressive function was seen after both 6 and 24 h pre-incubation, but strongest after 24 h pre-incubation (figure 3A and 3B). In the same experiment, TNFα expression was also suppressed in a dose dependent manner by dexamethasone, (figure 3C and 3D). After 6 h pre-incubation a weak suppression of TNFα secretion was seen for cocktail 4, 6, 7 and 8, but after 24 h pre-incubation the suppression was stronger. The suppressive effect of dexamethasone on cocktail 3 induced TNFα secretion was less prominent due to the addition of TNFα as a component of cocktail 3. However, cocktail 3 is useful for screening using other end-points like IL-12p70 and other cytokines, chemokines, maturation markers or ability to induce a MLR.

### Cocktail stimulated Prostaglandin E2 secretion and its suppression using unspecific and specific COX inhibitors

**Figure 4:**
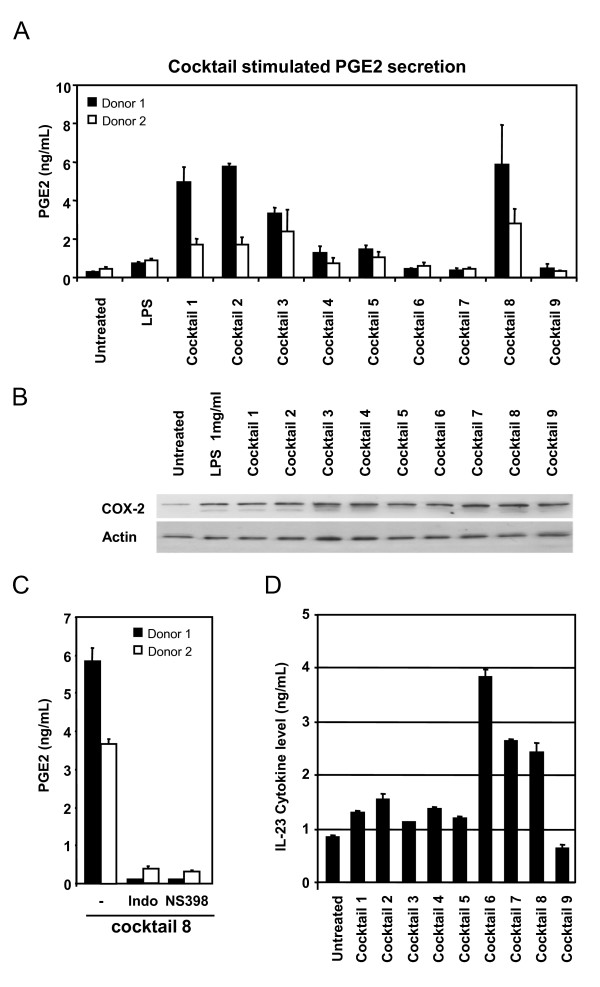
**Cocktail induced stimulation of inflammatory markers**. Prostaglandin E2 was determined in conditioned media from two representative donor derived DC batches as described in M&M (A). PGE2 secretion was highest when imDCs were treated with cocktail 1, 2, 3 and 8 for 24 hours. B) COX2 protein levels were analysed by western blot of lysates from DCs treated with cocktails for 24 h. Actin was used as loading control, and results shown are representative blot from two donors. C) Inhibition of PGE2 secretion induced by cocktail 8 was shown by pre-incubation of imDCs from two different donors for 2 h with the unspecific COX inhibitor indomethazine (indo) at 10 μM or the specific COX-2 inhibitor NS398 at 10 μM. D) The ability of cocktails to induce secretion of IL-23, shown as an average of measurements on 3 different donors. Bars show standard deviation between the three donors.

Prostaglandin E2 (PGE2) is a well known mediator of inflammation, and its secretion from dendritic cells treated with cocktails could serve as a relevant end-point in screening of anti-inflammatory compounds specifically targeting DCs. We determined the cocktail induced PGE2 secretion into the conditioned media from two different imDC batches. PGE2 secretion was low or weakly induced by LPS and cocktail 4, 5, 6, 7 and 9, whereas DCs treated with cocktail 1, 2, 3 and 8 showed higher PGE2 secretion (figure 4A). The higher levels of PGE2 stimulated by cocktail 1, 2, 3 and 8 was reflected in increased expression of COX2 compared to untreated cells, however, cocktails which did not stimulate PGE2 secretion to levels above untreated cells like LPS and cocktail 6, 7 and 9 also caused COX2 induction, shown by western blot of total lysates from DCs treated with the respective cocktails (figure 4B). The unspecific COX-1 and 2-inhibitor indomethazin, and the specific COX-2 inhibitor NS398 were both able to inhibit the secretion of PGE2 into the conditioned media, when added to the imDCs 2 h prior to addition of the most potent PGE2 stimulating cocktail 8 (figure 4C). Indomethazin and NS398 were not able to influence the DC mediated secretion of IL-6, TNFα or IL-12p70 (data not shown) into the conditioned media, indicating that these three cytokines are not induced in a PGE2 autocrine fashion, and that the drugs did not influence cell survival leading to decreased PGE2 production. Hence, the screening model is able to identify immuno modulating compounds which can influence COX-activation and PGE2 generation.

### Cocktail induced IL-23 secretion

IL-23 is a cytokine known to be involved in sustainment of Th17-typer responses implicated in chronic autoimmunity, and in particular IBD [7,8] and psoriasis [11-13]. We analysed the ability of the nine cocktails to induce secretion of IL-23 into the conditioned media, and found that the TLR7/8 agonist R848 was able to induce the IL-23 heterodimer (figure 4D). R848 induced IL-23 more than 4 fold above levels for untreated cells (figure 4D, cocktail 6), and when R848 was combined with IFNγ (cocktail 7) or poly I:C (cocktail 8) the IL-23 secretion was reduced. The remaining cocktails except cocktail 9, induced IL-23 moderately above the level seen for untreated cells.

### Maturation of cocktail treated DCs

**Figure 5:**
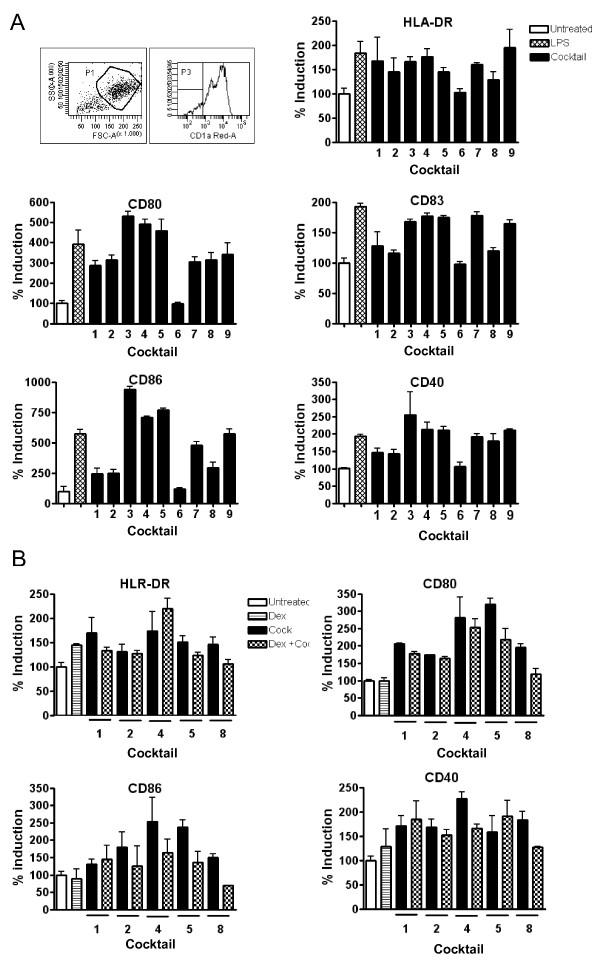
**FACS profile on cocktail stimulated DCs**. Surface staining by flow cytometric analysis of immature (untreated), LPS and cocktail stimulated DCs. The surface expression of relevant activation markers was analyzed on day 7. A) A total of 5000 events were collected by gating hDCs defined by forward (FSC) and side-scatter (SSC) characteristics. All histograms were gated for CD1a cells (70-95%). All our cells were CD14 negative (data not shown). Flow cytomeric analysis of maturation markes were done for DCs from three donors stimulated with LPS or cocktails, and results normalized to the untreated DCs (average value of Mean florescence intensity for untreated DCs from the three donors was set to 100%). The vertical bars indicate standard deviation (SD) values. B) Phenotypic surface analysis of the suppressive effect of dex on cocktail treated human DCs from two donors. Pre-treatment of immature DCs with dex for 6 h before addition of selected cocktails reduced the expression of activation markers. Results for the different treatments have been normalized in proportion to the untreated DC. The vertical bars indicate (SD) values.

A phenotypic analysis was performed by flow cytometry in order to investigate the expression of relevant maturation markers on the DCs after LPS and cocktail stimulation. LPS and all cocktails except cocktail 6 induce a high expression of the activation markers CD40, CD80, CD83, CD86 and HLA-DR (figure 5A). Selected cocktails with ability to stimulate these markers potently were selected for validation using dexamethasone pre-treatment for 6 h prior to addition of cocktail. Dexamethasone was able to lower the expression of a majority of the activation markers HLA-DR, CD40, CD80 and CD86 induced by the cocktails (figure 5B). The strongest effect of dexamethasone was found for cocktail 8 as the expression of activation markers CD80 and CD86 was found to be below the immature state.

### Allogeneic T-cell proliferation induced by cocktail treated DCs

**Figure 6:**
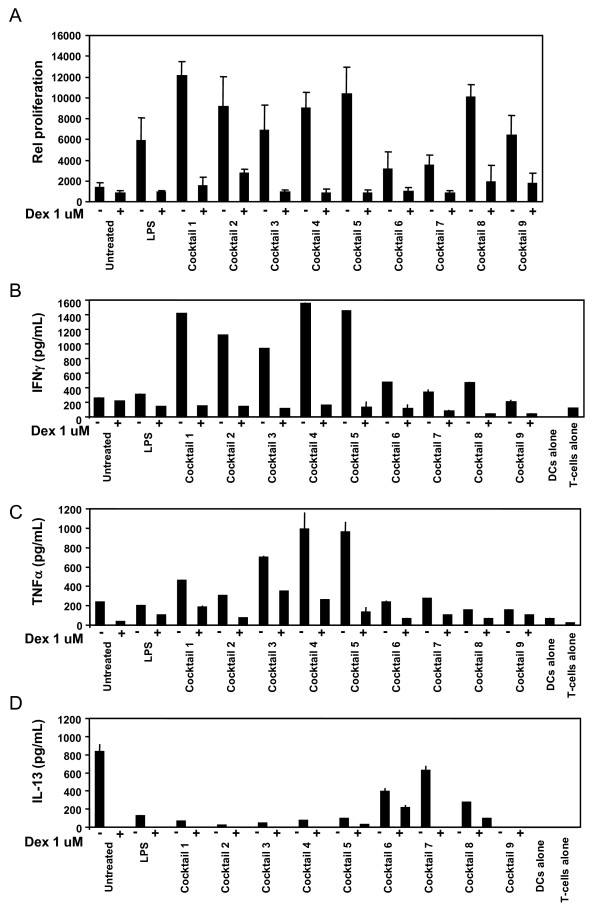
**T-cell proliferation and secreted cytokines stimulated by cocktail treated DCs**. MLR performed on CD4^+ ^T cells and allogeneic DCs. Mature cocktail stimulated DCs were more potent inducers of T cell proliferation in the MLR than immature DCs. A pretreatment of DCs with dexamethasone for 6 h before addition of cocktails, significantly prevent CD4^+ ^T cell proliferation to a level similar to immature DCs. The results are representative of three donors. A) CD4^+ ^T cells were cultured with allogeneic DCs for 5 days, and mitomycin-C treated in order to inhibit their proliferation. Proliferation of CD4^+ ^T cells was determined in the last 18-24 h of culture. Each column represents the mean cpm of four replicates. Vertical bars represent the SD. B) The amount of IFNγ production by T cells was measured in the supernatants after a restimulation with platebound anti-CD3 and soluble anti-CD28 mAbs for 24 h. In the conditioned media was also measured C) TNFα, and D) IL-13 by ELISA. Each column represents the mean of triplicate wells. Vertical bars represent the SD values.

The mixed lymphocyte reaction (MLR) was used as a functional endpoint to assess the *in vitro *T lymphocyte proliferation in response to DCs treated with the 9 cocktails. Cocktail treated DCs and CD4^+ ^T cells from allogeneic individuals were mixed together in a one-way primary MLR and T cell proliferation was measured by incorporation of ^3^H-thymidine (figure 6A). DCs were treated with cocktails alone or pre-treated with dexamethasone 6 h prior to addition of the cocktail. As seen in figure 6A, LPS and all cocktails stimulated T-cell proliferation, although cocktail 6 and 7 only stimulated approximately 2 fold higher proliferation than untreated cells. Pre-treatment of DCs with dexamethasone was able to suppress proliferation for all cocktails and LPS.

### Cocktail treated DC with ability to induce IFNγ producing T-cells

The ability of cocktail treated DCs to induce T-cell proliferation shows that these DCs have become immunogenic (figure 6A). However, increased proliferation does not indicate if the T-cells has differentiated into Th1, Th2 or Th17 T-cells when the DCs are cocultured with CD4^+ ^T cells. In order to evaluate whether the cocktails were truly Th1-inducing, we measured IFNγ in the conditioned media from T-cells restimulated with anti-CD3 and anti CD28. Cocktails 1-5 all induced high levels of IFNγ secretion, whereas LPS and cocktail 9 did not induce IFNγ above the level seen for untreated cells. Cocktail 6-8 induced IFNγ slightly above the control level (figure 6B). All cocktails showed reduced IFNγ secretion when the DCs were pretreated with dexamethasone as expected from the proliferation data. Cocktail 3, 4 and 5 were potent inducers of T-cell secreted TNFα, whereas the other cocktails were less potent in T-cell stimulated TNFα (figure 6C). Finally, in order to exclude that the cocktails induced a Th2 response, IL-4, IL-5 and IL-13 were measured in the T-cell conditioned media. IL-13 was highest in the media from untreated control cells, showing that in a MLR reaction by itself, a certain pool of the T-cells develop into Th2-cells. However, none of the cocktails induced IL-4, IL-5 or IL-13 above the level seen for untreated cells, showing that none of the cocktails induce a Th2-response (figure 6D, showing IL-13 as representative, IL-4 and IL-5 data not shown).

## Discussion

During recent years the role of DCs in immune disorders has been substantiated and the potential targeting of DCs for treatment of autoimmune and allergic diseases been suggested [14]. One of the interesting advantages of targeting DCs is their role as key initiators of adaptive immunity, thereby positioned upstream of the effector cells in e.g. autoimmunity [1,8]. Furthermore DCs have a relatively short life span compared to other primary cell types. At steady state conditions, immature DCs are quiescent until approached by a pathogen invading the tissue, or until tissue factors like cytokines or chemokines stimulate DC activation [2]. At sites of inflammation chemoattractants are produced by immune and epithelial cells, and will promote immature DC migration to the site of inflammation. After exposure to maturation stimuli at the inflammatory site, likely through stimulation with inflammatory chemokines and cytokines and/or combined with TLR agonists present at the site, DCs migrate to the lymph nodes and activate the adaptive immune response, and subsequently undergo apoptosis once they have activated a number of T-cells [15,16]. Thus, DCs at inflammatory sites have a higher flux through the inflammatory tissue.

We have approached the use of dendritic cells in targeting of immune disorders by establishment of a DC based *in vitro *screening model by mimicking this *in vivo *function of DCs. Immature DCs are developed from monocytes using conventional methods. The treatment with a drug candidate in our setting correlates to the potential treatment at the monocyte or steady state immature DCs level in the periphery of the patient, prior to chemoattraction of these cells from the circulation into the site of inflammation. The treatment in our *in vitro *model with cocktails containing Th1 inducing or inflammatory cytokines and TLR agonists mimics the *in vivo *situation where immature DCs are matured by factors at the site of inflammation. The cocktail matures the DCs towards a Th1-inducing phenotype, which mimics the maturation seen in many autoimmune conditions. By measuring drug induced changes in expression of maturation markers and secreted cytokines and chemokines, inflammatory lipids, intracellular signalling molecules and ability to induce T-cell responses, the effect on drug treated DCs *in vivo *can be predicted.

An interesting feature of DCs is their relatively complex expression of pattern recognition receptors and their corresponding signalling pathways. Some of the PRRs like e.g. TLR4 are expressed on antigen presenting cells in general, including monocytes, macrophages and B-cells, however, other TLRs are mainly or exclusively expressed on myeloid or plasmacytoid DCs. Monocyte derived myeloid DCs are known to express TRL1-11, except TLR9 which is expressed in plasmacytoid DCs [17,18]. TLR ligation has shown to be involved in several autoimmune diseases, where increased TLR ligands are present at the diseased site as well as in patient serum. One example is seen in patients suffering from RA, where TLR3, 4 and 7/8 expression is increased in the synovium, and where TLR ligation further encreases expression of inflammatory mediators in DCs from the RA patients compared to DCs from healthy controls [19]. We have in our present Th1-inducing cocktails utilized TLR ligation with ligands towards these 4 TLRs, by combining poly I:C (TLR3), LPS (TLR4) and R848 (TLR7/8) with proinflammatory cytokines in order to mimic the stimuli from autoimmune conditions.

The unique expression pattern of TLRs on antigen presenting cells and in particular on DCs, supports the idea that DCs are promising therapeutic target cells for treatment of inflammation and autoimmune disorders, since TLRs and their corresponding signalling pathways can be explored for more diverse target molecules, and in some cases targets that are exclusively expressed on DCs [10,20].

The cocktail treated DCs express intracellular inflammatory proteins like COX2, and membrane associated markers involved in regulation of adaptive immunity like HLA-DR, CD40, CD80, CD83 and CD86. Five of the defined cocktails potently stimulated development of Th1-cells, shown by their secretion of IFNγ in an allogeneic MLR.

Cocktail 1 was designed as in a previous reported cocktail [21], where the combination of poly I:C, IFNα, IFNγ, TNFγ and IL-1β showed very potent IL-12p70 stimulating properties compared to the standard DC cocktail used for DC based cancer vaccines, containing IL-6, TNFα, IL-1β and PGE2. The latter cocktail is slightly more potent in inducing DC migration, which is important for the DCs to reach local lymph nodes. In contrast, cocktail 1 was superior in inducing CTL-mediated cancer cell lysis and *in vivo *tumor antigen responses [21]. Our data show that cocktail 1 indeed induces PGE2 in itself, which could account for the migratory capabilities of cocktail 1 treated DCs as shown by Milliard and colleagues [21].

Our analyses of the 9 different cocktails in 15 donors showed that cocktail 3, 6 and 9 showed the greatest donor variation in IL-12p70 secretion. Although all cocktails induced significantly higher IL-12p70 secretion than LPS, the level of significance was lower for cocktail 6 and 9. The variation on TNFα secretion was slightly lower, where all cocktails and LPS significantly induced TNFα secretion compared to control treated cells, and with cocktail 1, 2, 3, 4, 5 and 8 significantly higher than LPS.

In most donors cocktail 6, which contains R848, was partly impaired in stimulation of IL-12p70, with an average level at 5 ng/mL. Taken in consideration that R848 only slightly stimulate IFNγ in the MLR assay, and is the most potent stimulator of IL-23, R848 seems to be a good candidate for induction of differentiation to a DC phenotype with ability to induce naïve CD4^+ ^T cells towards the Th17 lineage [22]. The secretion of IL-23 by R848 was reduced when the Th1-promoting cytokine IFNγ and the TLR3 agonist poly I:C were added together with R848. In particular the IL-23 suppressing ability of IFNγ supports the finding, where hall-mark Th1-promoting cytokines have the ability to suppress the Th17 promoting phenotype, a potential cross-talk discussed extensively for inflammatory diseases particular for intestinal inflammation [6]. Our data show that LPS alone was able to stimulate secretion of only minor amounts of both IL-12p70, IL-23 and IFNγ compared to cocktail treated DCs, and in particular in comparison to the two LPS containing cocktails 4 and 5, which contains IFNγ and IFNγ and TNFα respectively. This finding clearly shows that the identified cocktails are superior to the use of LPS alone as an immunogenic stimuli, and that our present model is superior as a DC based screening model for screening og drug candidates with potential Th1-suppressive function.

Combining LPS with IFNγ in our model (cocktail 4) preferentially increased IL-12p70 and not IL-23, which is in contrast to the findings by Roses et al. [22]. This difference can be explained by the fact that our imDCs were matured using IL-4 and GM-CSF for the whole culture period, where presence of IL-4 for the last 24 h of incubation reduces IL-12p70 and IL-23 production [22]. The finding by Roses et al., strongly indicates that *in vitro *culture conditions for both DCs and T-cells is influencing the ability of the model system to induce IL-23 and IL-17. Surprisingly, R848 was the only TLR agonist/cocktail that did not stimulate maturation marker expression HLA-DR and CD40, 80, 83 and 86.

The lower capacity of cocktail 9 to stimulate IL-12p70 could be explained by the lack of a TLR agonist in this cocktail, and indicates that the presence of IFNγ is not enough to drive potent induction of IL-12p70. However, in presence of a TLR agonist like LPS, IFNγ was able to potently polarize the DCs towards IL-12p70 induction (LPS alone vs cocktail 4), which is in line with other observations [21,22]. LPS and all cocktails more readily induced TNFα secretion from nearly all donor derived DCs, although the variations were also high for some cocktails, in particular for cocktail 6, 8 and 9. The most suitable cocktails for a DC based screening model should preferably induce the chosen end-point in as many donors as possible. However, for the ability of a compound to suppress cytokine secretion, the observed donor variation is not critical unless a certain amount of donors do not respond at all, as seen for e.g. LPS induced IL-12p70. In this regard, LPS is not a suitable maturation factor if one wishes to identify IL-12p70 or IL-23 suppressing compounds.

The DC responses of the cocktails were correlated to the pattern of chemokines and cytokines seen in autoimmune diseases. Using cytokine antibody arrays and ELISA we showed secretion of hall mark cytokines and chemokines like TNFα, IL-6, IL-10, IL-12p70, IL-23, IL-8, CCL2, -4, -5, -8, -15, -20, and CXCL1, 5 and 10. Interestingly, the cocktail treated DCs also induced secretion of particular VEGF and for some cocktails also GM-CSF.

The mimicry of these cytokine and chemokine patterns with specific disease pathologies is complex, in particular since tissue samples from patients suffering from these conditions contains factors secreted by a broad repertoire of leucocytes, fibroblasts and epithelial cells, and not DCs alone. However, certain comparisons are striking, where cocktail induced factors are similar to the ones identified in the pathological conditions. Biopsies from Crohn disease patients show increased expression of cytokines like TNFα, IL-1β IL-6, IL-12p70, IL-23 [7,8] and in particular the IL-12/IL-23 subunit p40 seems to be important for disease development since monoclonal antibodies towards this subunit is able to reduce symptoms and expression of both IL-12p70 and IL-23, but also IL-6 and IL-17 [23]. Chemokines implicated in Crohns disease are numerous, some includes CCL2, 4, 5, 8 and 20, and CXCL1, 2, 3, 8, 10, which are expressed by several of our cocktail treated DCs [24]. To this end, the cocktails mimicking the early phases of Crohns disease are cocktails 1, 2, 4 and 5, since they induce key factors like TNFα, IL-6, IL-12p70, CCL2, 4, 8, CXCL1-3 and upon T-cell co-culture also IFNγ. However, the later and chronic phase of Crohns disease involves activation of the IL-23/IL-17 axis, which might be better represented using R848 in the cocktails, as seen for cocktail 6, 7 and 8 [7,8,25]. In psoriatic lesions, cytokines like IL-6, IL-12p70, IFNγ and TNFα are identified, together with chemokines like CCL2-5 and CXCL1 [12,13], which shows that several of the cocktails stimulate cytokines and chemokines that to a large extent overlap with the ones expressed in different autoimmune conditions.

The tolerance inducing cytokine IL-10 was modestly induced by all cocktails, as well as LPS alone. This is a paradox compared to the ability of the cocktails to induce high IFNγ secretion, in particular for cocktail 1-5. However, the fact that both pro-inflammatory and anti-inflammatory cytokines are produced in the same pool of T-cells, is most likely due to the fact that the pool of T-cells to some extent are heterogeneous, and cocktail 1-5 most potently skew the T-cells towards the Th1 response, whereas other cocktails stimulate a more heterogeneous response [22].

Similarly, the pathogenesis of psoriasis involves key Th1 and Th17 inducing cytokines like TNFα, IL-1β, IL-2, IL-6, IL-10, IL-12p70, IL-23, CCL2, 3, 4, 5, 20, CXCL1, 8, 9 and 10, many of which are induced by cocktail treated DCs. The question whether TLR agonists are involved in the pathogenesis of psoriasis is unclear, but the finding that a TLR7 agonist, imiquimod, induces psoriasis like plaques in mice indicates that TLR7 induction could play a role [26]. In this respect, R848 or alternative cocktails using imiquimod could be the basis for design of cocktails with an optimal mimicry of the immune pathology seen in psoriasis. In addition, plasmacytoid DCs (pDC) also seems to play an important role in psoriasis, pointing to a potential pDC-based screening platform which could be generated using both TLR7 agonists and inflammatory cytokines. Other autoimmune diseases like RA and multiple sclerosis similarly involves combinations of Th1 and Th17 responses, suggesting that our present cocktails induce DC-phenotypes mimicking responses of DCs seen in these diseases [27-29].

## Conclusion

Nine cocktails were identified with potent ability to stimulate DC development into Th1-promoting inflammatory DCs with slightly different profiles mimicking DCs in autoimmune conditions as seen in Crohns disease, psoriasis, multiple sclerosis and RA. In particular 5 cocktails were able to mature DCs into potent Th1-inducing DC phenotypes, which showed high secretion of IL-6, TNFα and IL-12p70. When cocultured with CD4^+ ^T-cells, the cocktail martured DCs were able to stimulate development of Th1-cells which possessed a high proliferative capacity, and induced high levels of the hall-mark Th1 cytokine IFNγ.

The present screening model provides a novel preclinical screening tool, where Th1-suppressing compounds can be examined at several stages of immune activation; 1) at the innate receptor level on DCs, 2) at the innate TLR mediated signalling response in DCs, 3) at the level of DC-T cell interaction at receptor level or secretion of inflammatory mediators, 4) at the T-cell level targeting intracellular signalling and secretion of inflammatory mediators.

## Competing interests

The authors discloses that part of the work described in the present manuscript has been filed for a patent application. Furthermore, Bioneer A/S provides DC based screening services for customers.

## Authors' contributions

SJ drafted the manuscript, designed and evaluated cocktails and made data analysis to figures 1, 2, 3, 4. MG prepared and analysed data for figures 5 and 6. All experiments were carried out with technical help from Trine Møller and Bente M. Tolstrup. SJ and MG both read, commented and approved the final manuscript.
